# Atrial Fibrillation Ablation in a Patient with Partial Anomalous Pulmonary Vein

**DOI:** 10.19102/icrm.2026.17107

**Published:** 2026-10-15

**Authors:** Asim Mohiuddin, Prajakta Phatak, Mustafa Dohadwala

**Affiliations:** 1 Baylor Scott & White The Heart Hospital – Plano, Plano, TX, USA

**Keywords:** Anomalous pulmonary vein return, atrial fibrillation ablation, pulse field ablation

## Abstract

A 68-year-old man with persistent atrial fibrillation presented for ablation. Computed tomography showed a partial anomalous pulmonary venous return with connection of the right upper/middle vein to the superior vena cava. Wide antral circumferential ablation with pulse field ablation was used to isolate the normal veins connecting to the left atrium. The left atrium posterior right wall (usual position of the right upper pulmonary vein) and also the anomalous vein connection in the superior vena cava were important targets for mapping and possible ablation. However, in this case, these areas did not show independent firing and did not require additional ablation.

**CLINICAL IMPACT:**

This is the first reported case of the use of pulse field ablation (PFA) in a patient with partial anomalous pulmonary venous return. This case highlights the use of dense mapping in a congenital anomaly to avoid the risk of inadvertent injury to surrounding structures (in this case, injury to the superior vena cava). We hope to show this unique application of PFA ablation in a patient with a congenitally anomalous pulmonary vein and encourage operators to benefit from using newer technologies like PFA in patients with other congenital anomalies.

We present a 68-year-old man with persistent atrial fibrillation who was brought to the electrophysiology laboratory for atrial fibrillation ablation. He had a history of diabetes mellitus, hypertension, obstructive sleep apnea, and hypothyroidism. His primary cardiologist had started him on dronedarone and subsequently amiodarone.^1^ He required multiple direct-current cardioversions by the time he presented for electrophysiology consultation. With shared decision-making, ablation for persistent atrial fibrillation was pursued.

For procedural planning, a computed tomography of his heart was done, which revealed partial anomalous pulmonary venous return (PAPVR) of the right upper, middle, and superior portions of the lower lobe to the superior vena cava (SVC). His left-sided pulmonary veins were normal, and the remaining right-sided pulmonary veins drained via a right lower pulmonary vein into the left atrium (LA). He had not been previously diagnosed with this congenital anomaly **(Figure 1)**.

**Figure 1:**
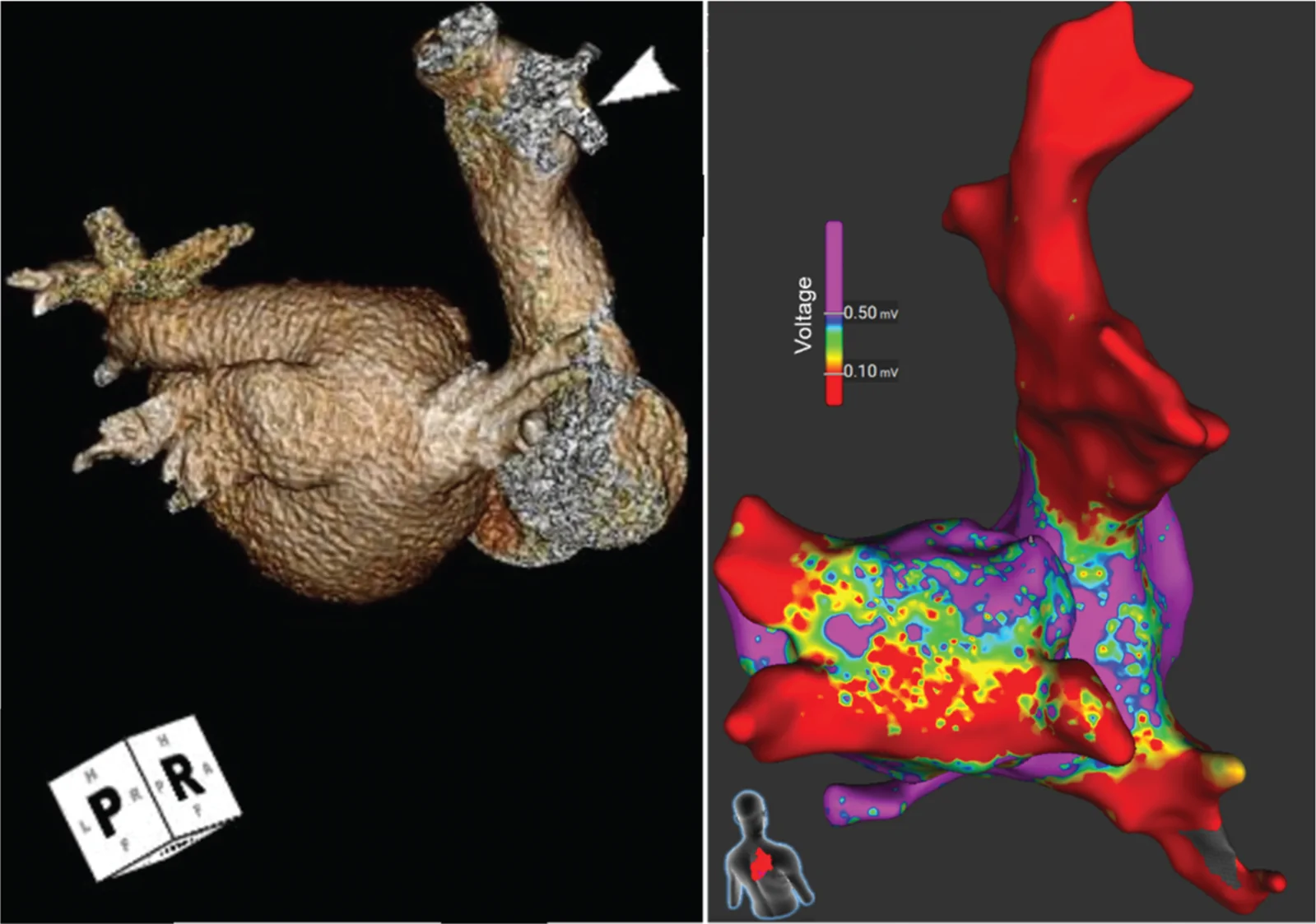
A computed tomography reconstruction and a dense electroanatomic mapping of both atria are shown, highlighting the anomalous pulmonary vein connection.

Our ablation approach for persistent atrial fibrillation is based on data from the Marshall Plan.^2^ This randomized controlled trial showed that pulmonary vein isolation and ablation of the roof line, typical flutter line, and inferolateral mitral line, followed by ethanol injection of the vein of Marshall, was superior to pulmonary vein isolation alone.^2^ We adjusted this lesion set to include pulmonary vein isolation and posterior wall, typical flutter line, and inferolateral mitral line ablation. We used a point-by-point pulsed field ablation (PFA) and radiofrequency ablation (RFA) combination lattice catheter for ablation of this lesion set (Sphere-9™; Medtronic, Inc., Minneapolis, MN, USA). Under this plan, PFA is performed for all lesions except for the cavotricuspid isthmus and mitral valve annulus, where RFA is used instead. If mitral block is not achieved, short PFA is performed in the coronary sinus adjacent to the endocardial mitral line. If that fails to achieve mitral block, we perform an ethanol injection into the vein of Marshall.

In this patient, the LA was mapped, and, subsequently, a wide antral circumferential ablation (WACA) lesion set was performed using PFA to isolate the left-sided pulmonary veins and the single, right-sided lower pulmonary vein. Then, posterior wall and mitral line ablations were performed **(Figure 2)**. The patient was then cardioverted and maintained a normal sinus rhythm. Both mitral block and entrance/exit block were demonstrated in the three pulmonary veins **(Figure 3)**.

**Figure 2:**
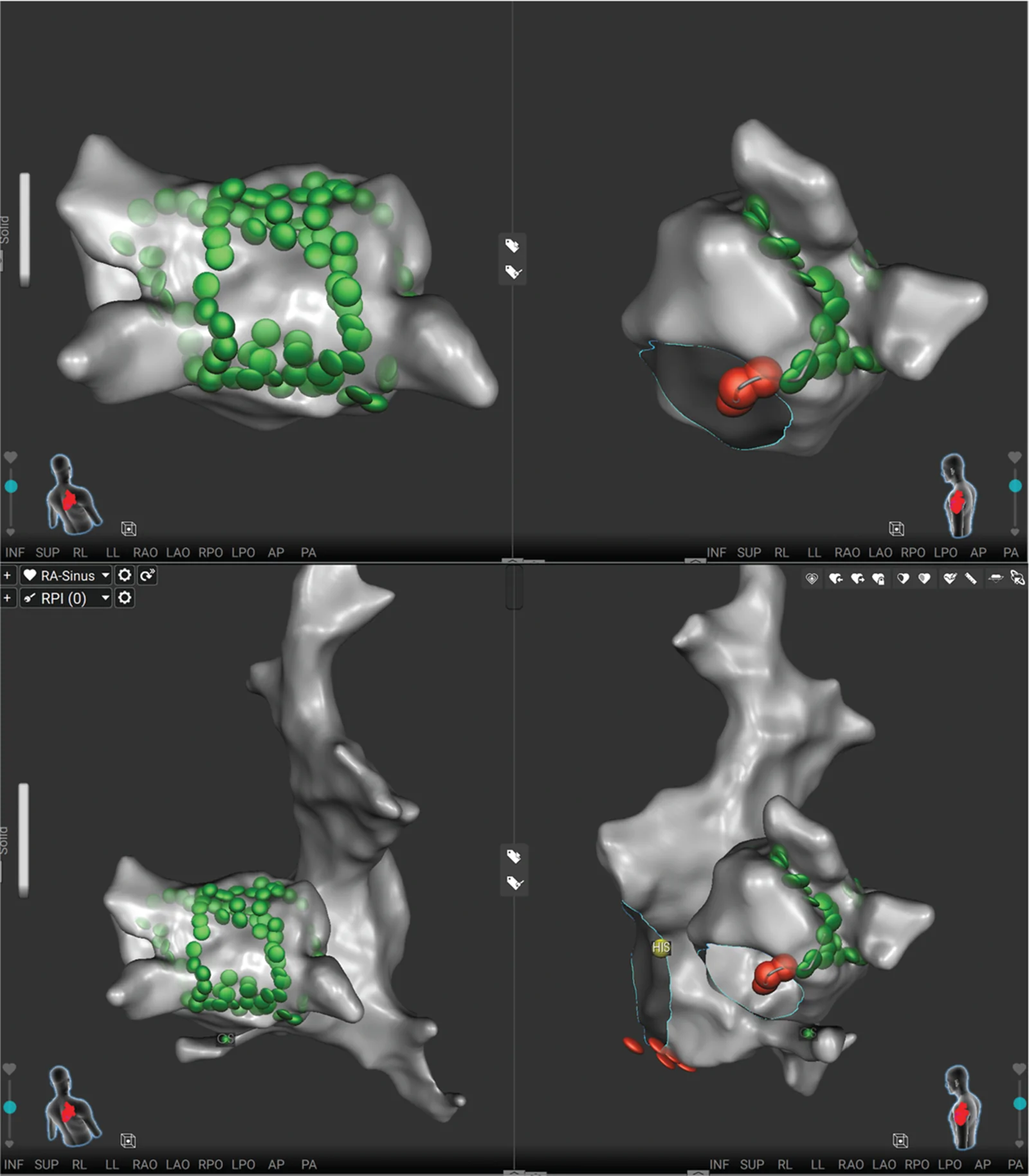
Electroanatomic map demonstrating the ablation lesion set tagged with green tags.

**Figure 3:**
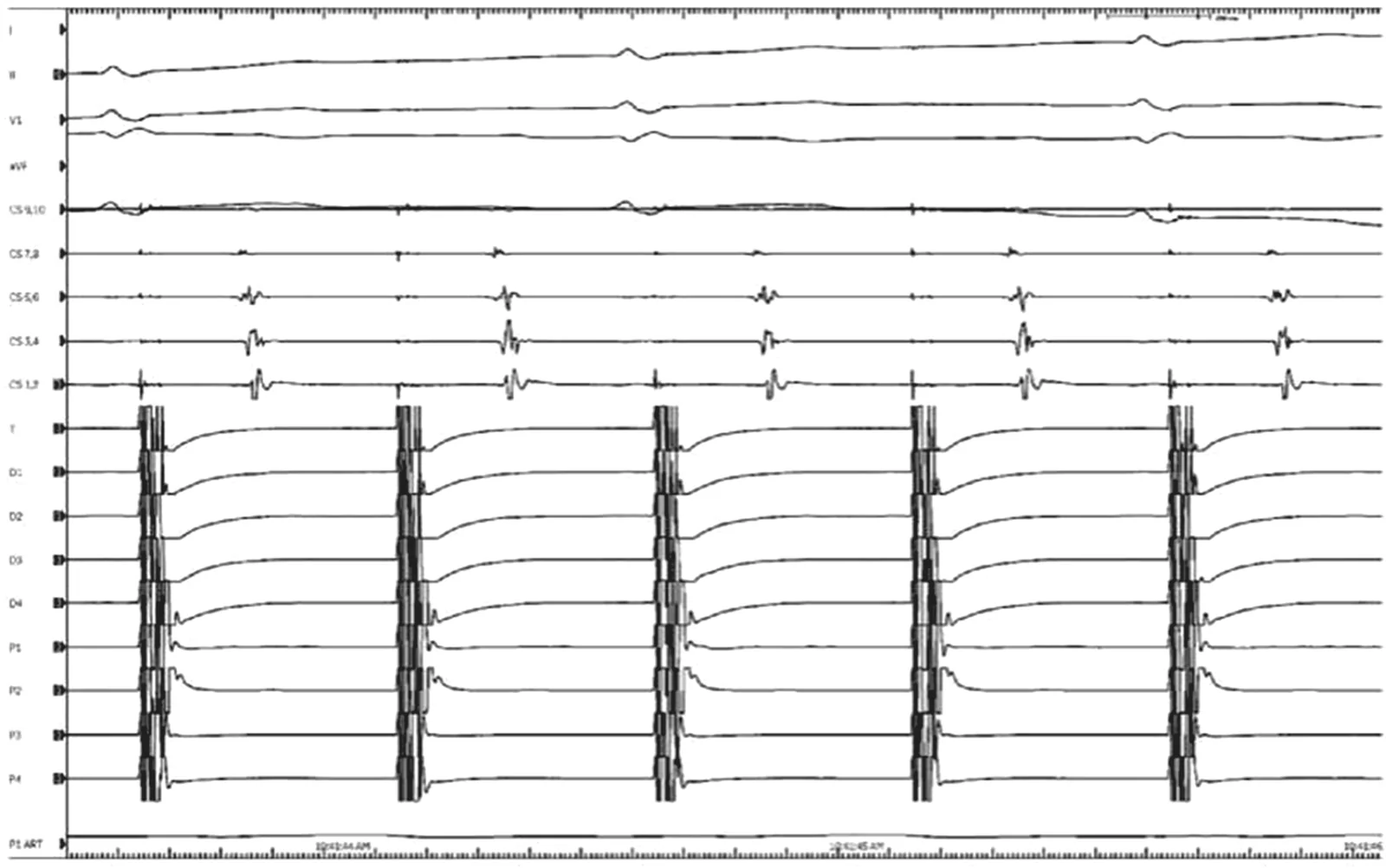
An intracardiac electrogram demonstrating mitral block.

At this point, we turned our attention to the right-sided anomalous pulmonary vein. We decided to use electroanatomic mapping (EAM) to further determine the need to isolate this vein. EAM of the right atrium, SVC, and anomalous pulmonary veins was performed. The 9-mm compressible, nitinol lattice-shaped catheter conformed well to the SVC and anomalous pulmonary vein. Also, with its nine mini-electrodes that are 0.7 mm in diameter, we were able to acquire 2962 voltage points within the SVC.^3^ With dense mapping, we were able to show that neither the anomalous pulmonary vein nor the SVC had any independent firing. Isoproterenol was then used to provoke any firing, but the anomalous vein remained electrically silent.

Finally, isolation of the SVC was also considered. SVC isolation may be a helpful adjunctive ablation strategy when there are documented triggers for atrial fibrillation. However, its benefit may be outweighed by potential complications (ie, phrenic nerve palsy, sinus node injury) when using thermal energies.^4,5^ PFA has the theoretical advantage of avoiding long-lasting phrenic nerve damage compared to thermal sources of energy such as RFA. We decided against SVC isolation for two main reasons. First, with detailed mapping—despite using isoproterenol—there were no premature atrial contractions from either the SVC or the anomalous vein. Second, despite the relative safety profile of PFA, there is still some inherent risk of injury to the sinus node during SVC isolation. We then completed our lesion set with typical flutter ablation. Three months’ post-ablation, a 2-week continuous monitor demonstrated sinus rhythm without recurrent atrial fibrillation off any anti-arrhythmic medications. He has had no symptomatic atrial fibrillation at his 6-month follow-up.

In conclusion, this is the first case reporting PAPVR during a PFA procedure using a lattice-shaped catheter. The dense voltage mapping allowed us to identify where the right upper pulmonary vein would have entered the LA to guide WACA and confirmed that the anomalous vein was truly silent. A case series involving 20 international centers has shown that PAPVR is rare. Only nine patients undergoing ablation were reported, and half were electrically silent.^6,7^ RFA and cryoballoon ablation were used in these cases. In one other published case, the EAM (CARTO™ map; Biosense Webster, Diamond Bar, CA, USA) did not include the right upper pulmonary vein.^6^ This case also highlights the decision-making behind SVC isolation required in PAPVR patients, since the anomalous pulmonary veins often connect to the SVC.^8^ In this case, SVC isolation was not performed, as neither the anomalous vessel nor the SVC had isolated potentials. We hope this case inspires the appropriate use of newer technologies for congenital anomalies and helps further expand the scientific front for these patients.
